# Targeted inhibition of *Mycobacterium tuberculosis* invasion into macrophages by Rv1768-Rv1983-loaded mannosylated chitosan nanoparticles

**DOI:** 10.3892/etm.2026.13337

**Published:** 2026-10-15

**Authors:** Jing Zhou, Mingwei An, Shidong Zhao, Yuwei Shan, Zhoubing Yu, Yun Xue, Fan Chen

**Affiliations:** 1 Wuhan Pulmonary Hospital (Wuhan Institute for Tuberculosis Control), Hubei Branch (Wuhan Pulmonary Hospital) of the National Clinical Research Center for Infectious Diseases, Wuhan, Hubei 430030, P.R. China; 2 State Key Laboratory of Biocatalysis and Enzyme Engineering, Department of Biochemistry and Molecular Biology, Life Science School of Hubei University, Wuhan, Hubei 430062, P.R. China; 3 Wuhan Biocause Pharmaceutical Development Co., Ltd., Wuhan, Hubei 430056, P.R. China; 4 Laboratory of Medical Engineering, College of Medical Technology and Engineering, Henan University of Science and Technology, Luoyang, Henan 471003, P.R. China

**Keywords:** *Mycobacterium tuberculosis*, Rv1768-Rv1983 fusion protein, mannosylated chitosan, nanoparticles, macrophage invasion

## Abstract

Tuberculosis (TB) remains a major threat to global health. The protective efficacy of the Bacillus Calmette-Guérin vaccine is limited because of the absence of multiple virulence-related genomic regions in *Mycobacterium tuberculosis* (MTB). The surface proteins of MTB are closely associated with bacterial invasiveness and pathogenicity, representing promising targets for novel vaccine development. In the present study, the MTB polymorphic GC-rich sequence proteins (Pro-Glu and Pro-Pro-Glu-Pro-Arg-Gly-Ser) family fusion protein Rv1768-Rv1983 was cloned, expressed and encapsulated into mannosylated chitosan (MCS) to construct MCS-Rv1768Rv1983 nanoparticles. The immunological and protective effects of this nanoformulation were evaluated *in vitro* and in a mouse model. Physicochemical characterization revealed that the nanoparticles had a mean particle size of 196.3 nm and a drug loading efficiency of 8.02%. *In vitro* cell experiments demonstrated that MCS-Rv1768-Rv1983 exhibited no obvious cytotoxicity and stimulated CD4^+^ T cells to secrete IFN-γ and IL-4, as well as CD8^+^ T cells, to produce IFN-γ. Animal experiments revealed that immunization with MCS-Rv1768-Rv1983 markedly elevated serum IgG1 and IgG2a titres and that the induced antibodies significantly reduced the invasion of virulent MTB H37Rv into macrophages. After H37Rv challenge, compared with the control group, the MCS-Rv1768-Rv1983 immunization group exhibited significantly lower bacterial loads in mouse lungs and markedly longer survival times. These results confirm that MCS nanoparticles can increase the immune efficacy of the Rv1768-Rv1983 fusion protein, block MTB invasion into macrophages and may contribute to the early clearance of MTB by macrophages.

## Introduction

According to the World Health Organization, tuberculosis (TB) affected 10.8 million people and caused 1.25 million mortalities in 2024(1). One-third of the global population has been infected with *Mycobacterium tuberculosis* (MTB), and 5-10% of these individuals develop active TB disease, with clinical symptoms such as a persistent cough, fever, night sweats and weight loss (2). Despite the widespread use of the Bacillus Calmette-Guérin (BCG) vaccine, its efficacy is limited in adults, partly because of the absence of immunogenic proteins (3). Therefore, the development and evaluation of an efficacious TB vaccine remains a major challenge.

Through comparative genomic hybridization between the MTB strain H37Rv and BCG, ~129 open reading frames were found to be distributed among H37Rv-specific genomic regions of deletion (RDs), designated RD1 to RD16 (4,5). These regions exist in H37Rv but not in BCG, suggesting that they may encode potential virulent antigens for bacterial pathogenesis and may be suitable for the development of new vaccine targets. TB vaccine candidates currently under study include mainly live attenuated vaccines, DNA vaccines, recombinant viral vector vaccines and protein subunit vaccines (6-8). Considering the potential risks of both whole-cell and virus-based vaccines, protein subunit vaccines appear to be more valuable for reagent application (9). However, the biggest problem with subunit vaccines is their insufficient immunogenicity. Therefore, it is important to optimize the vaccine components to trigger a stronger and more lasting immune response. Subunit vaccines formed by fusing immunodominant antigens from different stages of TB infection are beneficial for enhancing immunogenicity. Moreover, new vaccine adjuvants can prolong the immune stimulation response (10,11).

In recent years, chitosan-based nanoparticles have attracted considerable attention as safe and effective vaccine delivery systems (12,13). Chitosan is biocompatible, biodegradable and has low toxicity, and its cationic nature allows for efficient encapsulation of protein antigens. Furthermore, surface modification with targeting ligands, such as mannose, enables active delivery to antigen-presenting cells (APCs) via mannose receptors, thereby enhancing antigen uptake and cross-presentation. These properties make mannosylated chitosan (MCS) an ideal platform for subunit vaccines, especially against intracellular pathogens such as MTB.

Pro-Glu (PE) and Pro-Pro-Glu (PPE) family proteins serve increasingly important roles in the pathogenesis and chronic protracted infection observed in TB (14,15). The PE/PPE proteins of mycobacteria, defined by highly conserved N-terminal PE or PPE motifs, represent a unique and highly diverse group of proteins (16). The PE/PPE protein family constitutes 7-10% of the MTB genome and serves key roles in both bacterial physiology and host-pathogen interactions. They are involved in nutrient uptake, metabolism, enzymatic activity and drug resistance (17,18). PE/PPE proteins also modulate processes such as phagosome maturation, antigen presentation, autophagy, cell death regulation and immune invasion (19). In addition, PE/PPE proteins are rich in T-cell and B-cell epitopes, eliciting strong immune responses and thus representing promising candidates for subunit vaccine development (20). Different PE/PPE proteins may serve different roles in modulating host immunity, and the flexible interactions between these proteins contribute to their functional diversity, adding to the complexity of their study. Vaccines targeting PPE proteins, such as M72/AS01E and ID93/GLA-SE, have shown promise in clinical trials (21,22). MTB infects macrophages and persists in human macrophages for a prolonged period by escaping the host immune defence system (23). Preventing MTB from invading macrophages aids in the early clearance of bacteria and inhibits their colonization and proliferation. This may represent a novel approach for the development of TB vaccines.

In the present study, two proteins from the PE/PPE family were selected, namely, Rv1768 and Rv1983, and were combined to form a fusion protein. The fusion protein was subsequently prepared into MCS nanoparticles. The immune effects of the fusion protein nanoparticles were verified both *in vitro* and *in vivo*, especially their ability to inhibit the invasion of MTB into macrophages.

## Materials and methods

### Bacteria, cells and mice

*Escherichia coli* DH5α and BL21 (Thermo Fisher Scientific, Inc.), RAW264.7 macrophages (Mouse; ATCC), THP-1 cells (Human; ATCC), the pET32a plasmid (Novagen; Merck KGaA), and the virulent MTB H37Rv (ATCC 93009) and its genome were preserved. H37Rv was cultured in 7H9 Middlebrook broth (cat. no. 271310; Becton, Dickinson and Company) supplemented with 10% oleic acid, albumin, dextrose and catalase (Becton, Dickinson and Company). THP-1 cells were differentiated with 100 ng/ml phorbol 12-myristate 13-acetate (Sigma-Aldrich; Merck KGaA) for 48 h before the experiments. Female C57BL/6 specific pathogen-free mice at 6-weeks-old were purchased from the Hubei Center for Disease Control and Prevention. The mice were maintained under infection barrier conditions in a negative-pressure animal room and fed a sterile commercial mouse diet. All the studies were reviewed and approved by the Animal Ethics and Welfare Committee of Wuhan Pulmonary Hospital (approval no. 2024006).

### Cloning of the Rv1768 and Rv1983 genes and construction of the vector

The DNA sequences used in the present study were obtained from the GenBank database (accession no. NC_000962.3). All sequences were verified by Sangon Biotech Co., Ltd. sequencing to ensure accuracy. The amplification primers for Rv1768 (F: 5'-AAATGGGATGCCCGGGCCGCTCGAGCACCACCACCAC-3'; R: 5'-AGAAATGACACACCAGCACCGGCGCCC-3') and for Rv1983 (F: 5'-ATGGGTCGCG GATCCGTGTCATTTCTGGTCGTGGTTCC-3'; R: 5'-GCTTCCGCCACCGCCGCTTCCCGCCGGATGATCAAAGACT-3') were synthesized by Sangon Biotech Co., Ltd. PCR was performed using PrimeSTAR Max DNA Polymerase (Takara Bio, Inc.) under the following conditions: Initial denaturation at 95˚C for 5 min; 30 cycles of 95˚C for 10 sec, 55˚C for 15 sec and 72˚C for 1 min; and a final extension at 72˚C for 5 min. The PCR products were analyzed by 1% agarose gel electrophoresis and visualized by GelRed staining under UV light. Using the H37Rv genome as the template, the Rv1768 and Rv1983 genes were amplified, cloned and inserted into the pET32a vector by homologous recombination using a ClonExpress II One Step Cloning Kit (Vazyme Biotech Co., Ltd.) according to the manufacturer's instructions. The vector primers used for Rv1768 gene insertion were F: 5'-TGGTGTGTCATTTCTGGTCGTGGT-3'; R: 5'-TGGTGGTGGTGGTGCTCGAGCGGCCCGGGCATCC-3', whereas for Rv1983 gene insertion, the primers were F: 5'-GCCGTGAGAATTCGAGCTCCGTCGA-3'; R:5'-GCTTCCGCCACCGCCGCTTCCGGATCCGCGACCCA-3'. Finally, the expression vector pET32a-Rv1768/Rv1983 was constructed and transformed into *E. coli* DH5α competent cells. Briefly, 10 µl of the recombination reaction was added to 100 µl of competent cells, incubated on ice for 30 min, heat-shocked at 42˚C for 45 sec and placed on ice for 2 min. Next, 900 µl of LB broth was added, and the cells were recovered at 37˚C with agitation at 220 rpm for 1 h. The cells were plated onto LB agar containing 100 µg/ml ampicillin and incubated at 37˚C overnight.

### Bioinformatics analysis

The amino acid sequences encoded by Rv1768 and Rv1983 were retrieved from the NCBI database (accession no. NC_000962.3). Transmembrane helices were predicted using the TMHMM 2.0 server (DTU Health Tech, Technical University of Denmark) with default parameters (https://services.healthtech.dtu.dk/service.php?TMHMM-2.0).

### Expression and purification of fusion proteins

A single colony of BL21 cells carrying the expression vector was picked from an LB agar plate containing 100 µg/ml ampicillin and inoculated into 5 ml LB broth containing 100 µg/ml ampicillin. The culture was incubated overnight at 37˚C with agitation at 220 rpm. The overnight culture was then diluted 1:100 into fresh LB broth containing 100 µg/ml ampicillin and cultivated at 37˚C with agitation at 220 rpm until the optical density at 600 nm (OD600) reached 0.6. Subsequently, 0.5 mM isopropyl β-D-1-thiogalactopyranoside (Beijing Solarbio Science & Technology Co., Ltd.) was added, and expression was induced at 16˚C with agitation at 220 rpm for 18 h. The bacterial cultures before and after induction were collected, and the bacterial cells were harvested after induction. The bacterial cells were disrupted using an ultrasonicator (JY92-IIN; Ningbo Scientz Biotechnology Co., Ltd.) at a frequency of 20 kHz with 3 sec on/5 sec off cycles for 10 min in an ice bath, and then centrifuged at 12,000 x g for 30 min at 4˚C, after which the supernatant and precipitate were collected. The component containing the fusion protein was collected, and refolding was performed by sequential dialysis against renaturation buffer (50 mM Tris-HCl, pH 8.0, 0.5 M L-arginine, 5% glycerol, 1 mM dithiothreitol and 0.5 mM EDTA) containing decreasing urea concentrations [0.5 M for 6 h, then 0 M for 12 h with one buffer change, using a 10-kDa molecular weight cut-off (MWCO) dialysis membrane at 4˚C]. Afterwards, affinity chromatography on a nickel-nitrilotriacetic acid (Ni-NTA) column (GE Healthcare) was used for purification, and the target protein was eluted with 20, 80, 100, 150 and 300 mM imidazole solutions. Finally, proteins were separated by SDS-PAGE using a 12% separating gel and a 5% stacking gel, and then transferred onto a PVDF membrane (MilliporeSigma) at 100 V for 1 h at 4˚C. The membrane was blocked with 5% skimmed milk powder in TBST (20 mM Tris-HCl, pH 7.5, 150 mM NaCl and 0.1% Tween-20) for 1 h at room temperature. Next, membranes were incubated with a mouse anti-His tag monoclonal antibody (cat.no. ab18184; 1:2,000 dilution; Abcam) for 3 h at 4˚C, followed by three washes with TBST for 10 min each. Subsequently, the membrane was incubated with an HRP-labelled goat anti-mouse antibody (cat. no. 115-035-003; 1:5,000 dilution; Jackson ImmunoResearch Laboratories, Inc.) for 1 h at room temperature, followed by three washes with TBST for 10 min each. Protein bands were visualized using a DAB substrate kit (cat. no. DA1015; Beijing Solarbio Science & Technology Co., Ltd.) according to the manufacturer's instructions.

### Preparation of MCS nanoparticles

First, 0.25 g chitosan (Sigma-Aldrich; Merck KGaA) was weighed and placed in a 150 ml beaker and 50 ml 5% acetic acid aqueous solution (pH 5.0) was added to it. The beaker was then placed on a magnetic stirrer and stirred at 400 rpm and 40˚C for 1 h. Subsequently, 0.25 g mannose (Sigma-Aldrich; Merck KGaA) and 0.25 g sodium triacetoxyborohydride (Sigma-Aldrich; Merck KGaA) were weighed, dissolved in 10 ml pure water, and the mixed aqueous solution was added dropwise into the chitosan solution using a dropper. The mixture was stirred at room temperature for 24 h. The resulting liquid was transferred into a 10 kDa dialysis bag (Repligen) and dialyzed against pure water on a magnetic stirrer at room temperature for 3 days to obtain an aqueous solution of MCS. After vacuum freeze-drying, the MCS was ready for use. The fusion protein was mixed with the MCS solution in a beaker (1:10) and stirred at 500 rpm on a magnetic stirrer for 30 min. Then, 1 mg/ml tripolyphosphate sodium (TPP; Sigma-Aldrich; Merck KGaA) was slowly added dropwise, and the mixture was stirred for 1 h to obtain MCS-Rv1768-Rv1983. The optimal mass ratio of MCS to TPP (C/T), optimal pH and optimal stirring time were determined through single-factor experiments (Figs. S1 and S2).

### Particle size and appearance of nanoparticles

Potassium bromide was removed from the drying oven, placed under a tungsten filament lamp to dry, ground into powder form and mixed evenly with the sample.

For Fourier transform infrared spectroscopy (FTIR) analysis, the samples (MCS, chitosan and mannose) were ground with potassium bromide powder at a ratio of 1:100 and pressed into translucent pellets. FTIR spectra were recorded in the range of 4,000-400 cm^-1^ with a resolution of 4 cm^-1^ using a Nicolet iS10 spectrometer (Thermo Fisher Scientific, Inc.).

A particle size analyzer (ZS90; Malvern Instruments, Inc) was used to determine the particle size, polydispersity index and zeta potential of the nanoparticles at 25˚C. The nanoparticles were freeze-dried and dispersed into pure water. A pipette was used to drop the dispersed sample onto a sample carrier copper grid and allowed to air dry at room temperature. Then, the sample was placed under a transmission electron microscope (Libra 120; Zeiss AG) to observe the morphology of the nanoparticles.

### Drug loading capacity

The prepared MCS-Rv1768-Rv1983 solution was centrifuged at 13,684 x g for 30 min at 4˚C. The supernatant was collected, and the precipitate was resuspended in 1 ml of pure water to ensure complete resuspension. The resuspended precipitate was then placed in a freeze-dryer and dried for 24 h to form nanoparticle powder. The protein concentration of the nanoparticle solution before centrifugation and the concentration of unencapsulated protein in the supernatant were measured using a Nanodrop 8000 spectrophotometer (Thermo Scientific, Ltd.). The supernatant of the blank MCS nanoparticles was used as a control. Drug loading (DL; %) was calculated according to the following formula: DL (%)=[((C_t_-C_b_)-(C_s_-C_bs_)) x V/M] x100, where C_t_ is the protein concentration of the nanoparticle solution before centrifugation, C_b_ is the protein concentration of the blank nanoparticle solution, C_s_ is the protein concentration in the supernatant after centrifugation, C_bs_ is the protein concentration of the blank nanoparticle supernatant, V is the volume of the nanoparticle solution and M is the total mass of the dried nanoparticles.

### Release determination

The prepared nanoparticle solution was centrifuged at 13,684 x g for 30 min, after which the precipitate was collected, resuspended in 15 ml pure water, placed in a dialysis bag (10 kDa MWCO), sealed at both ends and immersed in 100 ml PBS (pH 7.4) on a magnetic stirrer at 37˚C and 100 rpm. Samples were taken at 0, 0.5, 1, 3, 6, 12, 24, 36, 48, 56 and 72 h. Each sample was replaced with an equal volume of fresh dialysis medium. The protein concentration at each time point was determined using the Nanodrop. The cumulative release rate Q was calculated as follows: Qn=C_1_V_1_ + C_2_V_2_ +…+ C_n_V_n_; Q=Qn/M x 100, where C_1_ and C_n_ are the sample concentrations at different time points; V_1_ and V_n_ are the sample volumes taken each time; Q_n_ is the cumulative release amount; and M is the total mass of protein in the nanoparticles.

### Cell cytotoxicity

The cytotoxic effects of the MCS nanoparticles on THP-1 cells were assessed using the Cell Counting Kit-8 Cell Proliferation and Cytotoxicity Assay Kit (cat. no. CA1210; Beijing Solarbio Science & Technology Co., Ltd.). Cells were cultured in 96-well plates at 5x10^4^ cells/well for 24 h at 37˚C in a 5% CO_2_ incubator, containing 200 µl RPMI-1640 medium supplemented with 10% FCS (Gibco; Themo Fisher Scientific, Inc.), 100 U/ml penicillin and 100 µg/ml streptomycin (Gibco; Themo Fisher Scientific, Inc.). The cells were treated with MCS, Rv1768-Rv1983 and MCS-Rv1768-Rv1983 at 50, 100, 150 and 200 µM for 24 and 48 h, respectively. PBS-treated cells served as controls. Cell viability was determined using an ELISA reader (K6600; Beijing Kaiao Technology Development Co., Ltd.).

### Animal model

A total of 24 female C57BL/6 mice were randomly divided into four groups (n=6 per group) as follows: Control group, injected with 200 µl PBS only; MCS group, 0.50 mg MCS dissolved in 200 µl PBS; Rv1768-Rv1983 protein group, 40 µg Rv1768-Rv1983 dissolved in 200 µl PBS; and MCS-Rv1768-Rv1983 group, 0.50 mg MCS encapsulating 40 µg Rv1768-Rv1983 dissolved in 200 µl PBS. Sample size was determined using a power analysis based on the Schoenfeld formula for the log-rank test, with a two-sided significance level of α=0.05, power=80% and an expected hazard ratio of 0.5. Each group was subcutaneously injected once at weeks 0, 2 and 4. Blood samples (~100 µl per mouse per time point) were collected from the orbital sinus using heparinized capillary tubes under isoflurane anesthesia (3-4% for induction, 1-1.5% for maintenance, by inhalation). The first collection was performed 2 weeks after the first immunization (week 2, for T-cell activity analysis), and the second collection was performed at 2 weeks after the last immunization (week 6, for antibody detection), with an interval of 4 weeks between the two draws. To minimize local tissue damage, the two collections were performed from the contralateral eye (left eye for the first draw, right eye for the second draw). Afterwards, the mice were challenged with 5x10^7^ colony-forming units (CFUs) of H37Rv via tail vein injection. Mice were monitored twice daily for signs of morbidity. The humane endpoints established for the present study included: i) ≥20% loss of initial body weight; ii) persistent lethargy, hunched posture or reluctance to move; iii) respiratory distress (labored breathing); or iv) inability to reach food or water. Any mouse meeting at least one of these criteria was immediately euthanized. During the experiment, none of 24 mice reached these humane endpoints. After all the mice in the control group died, all surviving mice were euthanized by cervical dislocation under deep anesthesia induced by intraperitoneal injection of pentobarbital sodium (150 mg/kg). Lungs and spleens were dissected and ground in 1 ml PBS, serially diluted 10-fold and cultured on Löwenstein-Jensen medium plates (BD Biosciences) at 37˚C for 4 weeks. MTB colonies were counted, and the results were expressed as log_10_ CFU per organ. Portions of lungs from three mice in each group were fixed with 4% paraformaldehyde for 24 h. The tissues were embedded in paraffin and 4-µm sections were cut. After deparaffinisation in xylene twice for 10 min each and rehydration through a graded ethanol series (100, 95, 80 and 70%; 5 min each), sections were examined via H&E and acid-fast staining (Ziehl-Neelsen method). H&E staining was performed with haematoxylin for 5 min and eosin for 2 min at room temperature. Ziehl-Neelsen staining was performed with carbol fuchsin for 10 min and methylene blue for 1 min at room temperature. Sections were observed under a Leica CW4000 microscope, and images were captured using Leica Microsystems GmbH.

### Cytokine analysis

Blood cells were isolated from the mice 2 weeks after the first immunization. After the red blood cells were lysed in ammonium chloride potassium buffer (Gibco; Thermo Fisher Scientific Inc.), 5x10^5^ peripheral blood mononuclear cells (PBMCs) isolated from mouse blood and cultured for 48 h in DMEM (Sigma-Aldrich; Merck KGaA) supplemented with 10% FCS, 100 U/ml penicillin and 100 mg/ml streptomycin. Single-cell suspensions were stimulated for 16 h at 37˚C and 5% CO_2_ using the fusion protein (5 µg/ml). The cells were continuously cultured for another 5 h in the presence of GolgiPlug (BD Biosciences) according to the manufacturer's instructions. Non-specific binding was blocked by incubating cells with 5 µl FcR blocking reagent (cat. no. 422302; BioLegend, Inc.) per 10^6^ cells in 100 µl staining buffer for 10 min at room temperature. The activated cells were then surface-stained with an FITC-labelled anti-CD4 antibody (cat. no. 340133; 1:20 dilution; BD Biosciences) and a PerCP-Cy5.5-anti-CD8 antibody (cat. no. 341050; 1:40 dilution; BD Biosciences). After surface staining, cells were fixed and permeabilized using the BD Cytofix/Cytoperm Fixation/Permeabilization Kit (cat. no. 554714; BD Biosciences) according to the manufacturer's instructions. Briefly, cells were resuspended in 250 µl BD Cytofix/Cytoperm solution and incubated for 20 min at 4˚C, followed by two washes with 1 ml BD Perm/Wash buffer (cat. no. 554723; BD Biosciences). The cells were then intracellularly stained with PE-anti-IFN-γ (cat. no. 340452; 1:50 dilution; BD Biosciences) and APC-anti-IL-4 (cat. no. 554436; 1:100 dilution; BD Biosciences) in BD Perm/Wash buffer. The numbers of specific cell types were quantified using a FACSAria flow cytometer (BD Biosciences), and data were analysed using FlowJo^™^ software version 10.10 (BD Biosciences).

### Analysis of antibody response

To measure the antibody levels of IgG1 and IgG2a, ELISA was used 2 weeks after the last vaccination, using recombinant Rv1768-Rv1983 fusion protein as a coating antigen. In brief, 2 µg/ml of the fusion protein was coated onto ELISA plates (Costar; Corning, Inc.) overnight at 4˚C in NaHCO_3_ buffer (pH 9.6). Next, 5% BSA (Sigma-Aldrich; Merck KGaA) in PBS was added for 2 h at 37˚C. Afterwards, the plates were incubated for 2 h with serum samples at a 1:8 dilution. Subsequently, the bound antibodies were detected with HRP-conjugated goat anti-mouse IgG1 and IgG2a (cat. nos. A2554 and A2664; 1:3,000; Sigma-Aldrich; Merck KGaA) for 1 h at 37˚C. Between each step, plates were washed three times with PBS containing 0.05% Tween-20 (200 µl/well) for 3 min each. After being washed, the assay was developed in o-phenylenediamine dihydrochloride-H_2_O_2_ substrate solution (Sigma-Aldrich; Merck KGaA) for 15 min at room temperature in the dark. The reaction was stopped by adding 50 µl of 2 M H_2_SO_4_ per well,and the absorbance was measured at 492 nm using a microplate ELISA reader (K6600, Beijing Kaiao Technology Development Co., Ltd.).

### Phagocytic index detection

RAW264.7 macrophages were cultured in RPMI-1640 supplemented with 10% FBS and penicillin/streptomycin. After 48 h, the cells were counted by Trypan blue staining. To evaluate the effects of antibodies against H37Rv invasion of macrophages, 1x10^6^ CFU of H37Rv were first incubated with serum (1:10) from different groups at 37˚C for 15 min. Then, the samples were centrifuged at 13,684 x g for 5 min at 4˚C, after which the supernatant was discarded. RAW264.7 macrophages (1x10^6^) were mixed with opsonized H37Rv in 2 ml medium. Phagocytosis was allowed to occur for 1 h at 37˚C in 6-well plates, each well containing a coverslip pre-treated with polylysine (0.1 mg/ml). RAW264.7 cells were seeded at a density of 1x10^6^ cells/well in 2 ml medium. The coverslip was removed, and the cells were fixed with ice-cold acetone at -20˚C for 10 min. The cells were then stained with auramine O (Sigma-Aldrich; Merck KGaA) for 15 min at room temperature and washed with PBS. Finally, the coverslips were observed under a fluorescence microscope (CW4000; Leica Microsystems GmbH). A total of 100 cells were counted from each coverslip. The phagocytic index was calculated as follows: Phagocytic index=total MTB captured by macrophages/number of macrophages capable of capturing bacteria.

### Statistical analysis

The experimental data were analysed with GraphPad Prism 10.1 (Dotmatics). For normally distributed data, comparisons among multiple groups were performed by one-way analysis of variance (ANOVA) followed by Student-Newman-Keuls (SNK) post hoc test. For the cytotoxicity assay, data at each time point (24 and 48 h) were analyzed separately using one-way ANOVA with SNK post hoc test to compare differences among concentration groups; no statistical comparison was performed between the two time points. Survival curves were analysed using the Kaplan-Meier method with the log-rank (Mantel-Cox) test. Mycobacterial counts were log_10_-transformed and analysed by one-way ANOVA. P<0.05 was considered to indicate a statistically significant difference.

## Results

### Expression and purification of the Rv1768-Rv1983 fusion protein

Fragments of the Rv1768 and Rv1983 genes were amplified separately, with sizes of 1,854 and 1,674 bp, respectively (Fig. 1A). Using homologous arm recombination, Rv1768 and Rv1983 were ligated into the pET32a vector. The two genes were connected by a 21 bp flexible linker composed of alternating glycine and serine residues. Successful construction of the fusion expression vector was confirmed by electrophoresis and sequencing (Fig. 1B). After the induction, the Rv1768-Rv1983 fusion protein, with a molecular mass of ~129 kDa, was expressed in the form of inclusion bodies (Fig. 1C). Purification was performed using a Ni-NTA affinity chromatography column, and the target protein was obtained with a relatively high purity under 150 mM imidazole gradient elution (Fig. 1D). Western blot analysis further confirmed the successful expression and purification of the fusion protein (Fig. 1E). Bioinformatics analysis revealed no transmembrane regions in Rv1983 and Rv1768, suggesting that they are both extracellular proteins (Fig. S1).

### Physical properties of the fusion protein nanoparticles

In Fig. 2A, the synthesis pathway of MSC is illustrated. FTIR analysis revealed characteristic absorption peaks at the hydroxyl stretching vibration at 3,376 cm^-1^, C-H stretching vibration at 2,927 cm^-1^ and N-H bending vibration at 1,650 cm^-1^, confirming the presence of primary amine groups. Additionally, the N-H bending vibration at 1,558 cm^-1^ and C-N stretching vibration at 1,380 cm^-1^ in the MCS indicated the formation of imine groups through reductive amination between mannose and chitosan, confirming the successful grafting of mannose onto chitosan (Fig. 2B). The optimal conditions for preparing MCS nanoparticles were determined as follows: MCS concentration of 1 mg/ml, TPP concentration of 1 mg/ml, C/T ratio of 5:1, pH of 5.0 and stirring time of 50 min (Figs. S2 and S3). The resulting MCS-Rv1768-Rv1983 nanoparticles had an average particle size of 196.3 nm, a polydispersity index of 0.297 and a zeta potential of -12.7 mV (Fig. 2C). When the concentration of the fusion protein was 0.4 mg/ml, the drug loading capacity was 8.02% (Fig. S4). Transmission electron microscopy revealed that the nanoparticles were elliptical in shape, uniform in morphology and well dispersed (Fig. 2D). The release profile of MCS-Rv1768-Rv1983 revealed an initial rapid release phase, reaching ~80% within 12 h, followed by a sustained release phase entering a plateau, with a cumulative release of 95% over 24 h (Fig. 2E). These data indicate that the encapsulation of the fusion protein by MCS provides a sustained release effect and efficient protein utilization.

### Effect of MCS on cell viability

Cytotoxicity analysis results revealed that the toxicity of MCS to THP-1 cells was extremely low. Compared with the PBS treatment group, after 24 h of interaction with different concentrations of MCS (50-200 µM), cell viability remained essentially unchanged (Fig. 3A). After 48 h, the cell viability remained >90% for all the tested MCS concentrations. Treatment with Rv1768-Rv1983 or MCS-Rv1768-Rv1983 for 24 and 48 h led to a moderate decrease in cell viability, but it remained >85% (Fig. 3B; P<0.05 for Rv1768-Rv1983 and P<0.01 for MCS-Rv1768-Rv1983 vs. PBS control). According to the ISO 10993-5:2009 guidelines for biological evaluation of medical devices, a cell viability of >70% is considered non-cytotoxic (24). The present results, showing cell viability consistently >85%, are well above this threshold.

### Cytokine production in PBMCs

To evaluate T-cell activation, the percentages of IFN-γ- and IL-4-producing CD4^+^ and CD8^+^ T cells in PBMCs were measured 2 weeks after the first vaccination. After *ex vivo* stimulation with the fusion protein for 16 h, the percentages of IFN-γ- and IL-4-producing CD4^+^ T cells were significantly greater in the Rv1768-Rv1983 and MCS-Rv1768-Rv1983 groups than in the control and MCS groups (Fig. 4A and B). One-way ANOVA with an SNK post hoc test revealed significant differences between the two immunization groups and the control groups (both P<0.01). For CD8^+^ T cells, the percentage of IFN-γ-producing cells was also significantly greater in the immunization groups than in the control groups (Fig. 4C; P<0.05), whereas the percentage of IL-4-producing CD8^+^ T cells was not significantly different among the groups (Fig. 4D; P>0.05).

### Antibody responses and inhibition of macrophage invasion

To determine the induced humoral immune response, serum IgG1 and IgG2a subclasses were analysed. Compared with those in the control and MCS groups, the IgG1 titres in both the Rv1768-Rv1983 and MCS-Rv1768-Rv1983 groups were significantly greater; moreover, compared with the Rv1768-Rv1983 group, the MCS-Rv1768-Rv1983 group exhibited higher IgG1 levels (Fig. 5A; P<0.05 for MCS-Rv1768-Rv1983 vs. Rv1768-Rv1983). IgG2a levels were also significantly higher in both the immunization groups than in the control and MCS groups, but no significant difference was found between the Rv1768-Rv1983 and MCS-Rv1768-Rv1983 groups (Fig. 5B; P>0.05). After different anti-sera were incubated with H37Rv, the ability of the bacteria to invade macrophages was notably altered. Compared with the Rv1768-Rv1983 group, the MCS-Rv1768-Rv1983 group had a significantly lower phagocytic index (Fig. 5C; P<0.01), indicating that MSC encapsulation enhances antibody-mediated inhibition of MTB invasion.

### In vivo protective efficacy

Survival analysis revealed that compared with the control group, the MCS-Rv1768-Rv1983 group had significantly longer survival (log-rank test, P<0.05), whereas the survival rate of the Rv1768-Rv1983 group did not significantly change (Fig. 6A; P>0.05). Bacterial loads in murine lungs and spleens were determined at 4 weeks after sacrifice. Compared with the control groups, bacterial counts in the lungs were significantly lower in both the MCS-Rv1768-Rv1983 (P<0.01) and Rv1768-Rv1983 groups (P<0.05), while compared with the MCS group, bacterial counts in the lungs were significantly lower in the MCS-Rv1768-Rv1983 group (P<0.05) (Fig. 6B). Notably, compared with the Rv1768-Rv1983 group, the MCS-Rv1768-Rv1983 group exhibited a stronger inhibitory effect on bacterial proliferation. In the spleen, the bacterial counts did not significantly differ among the groups (Fig. 6C; P>0.05). Histopathological examination of lung sections revealed that the control group had marked inflammatory reactions, including alveolar consolidation and fusion. By contrast, both the MCS-Rv1768-Rv1983 and the Rv1768-Rv1983 groups showed less alveolar fusion, reduced swelling and more prominent air spaces (Fig. 6D). Acid-fast staining also revealed fewer acid-fast bacilli in the lungs of MCS-Rv1768-Rv1983-immunized mice than in those of control mice (Fig. 6D; upper panels).

## Discussion

The immune evasion mechanism of MTB has not been fully elucidated. However, if the invasion of MTB into macrophages is prevented, the host immune system may be able to clear the bacteria at an early stage (25). With the continuous deepening understanding of MTB pathogenicity, a number of new vaccine candidates have been discovered and some are already in preclinical trials aiming to replace or enhance the efficacy of BCG (26). Among the TB vaccine candidates currently in clinical trials, protein subunit formulations serve a pivotal role. However, optimizing antigen combinations is important for overcoming the limited immunogenicity of protein subunit vaccines (27).

The present successful cloning, expression and purification of the Rv1768-Rv1983 fusion protein provides a solid basis for its evaluation as a vaccine antigen. Inclusion body formation and subsequent refolding by gradient dialysis ensured adequate protein yield and purity. Furthermore, the physicochemical characterization of MCS-encapsulated nanoparticles revealed favourable properties, an average size of ~200 nm, a negative zeta potential and a sustained release profile, which are known to favour lymphatic drainage and antigen presentation. The excellent biocompatibility of the MCS confirms its safety as a vaccine carrier, supporting its use in subsequent *in vivo* studies. Rv1768 belongs to RD14 and can induce IFN-γ production by mouse PBMCs, as well as the generation of specific IgG and IgM against Rv1768; hence, it is a promising candidate antigen (28). Studies have also shown that Rv1768 promotes MTB survival in macrophages by regulating the NF-κB-TNF-α signalling pathway and by modulating arachidonic acid metabolism through S100A9 (29,30). Rv1983 is an immunodominant antigen that belongs to RD2. Rv1983 can induce IFN-γ responses and has been identified as a potential diagnostic agent or subunit vaccine candidate (31,32). In the present study, the fusion of these two antigens elicited robust T-cell responses.

In addition to the cellular immune responses dominated by IFN-γ, the present data revealed a marked increase in the level of IL-4 secreted by CD4^+^ T cells in mice immunized with Rv1768-Rv1983 and MCS-Rv1768-Rv1983. IL-4 is a signature Th2 cytokine that promotes B cell activation, antibody class switching and the production of IgG1, which is consistent with the robust IgG1 response observed in the present study. The balanced induction of both IFN-γ (Th1) and IL-4 (Th2) indicates a mixed Th1/Th2 immune profile, which is favourable for protective anti-TB immunity through the coordination of cellular clearance and humoral neutralization. Previous studies have confirmed that Th2 responses support antibody-mediated inhibition of mycobacterial invasion into macrophages, thereby complementing Th1-dependent bactericidal activity (33-35). Thus, the upregulation of IL-4 further supports the notion that MCS-encapsulated Rv1768-Rv1983 induces a well-balanced protective immune response rather than a polarized Th1 or Th2 reaction.

A major novelty of the present work is demonstrated by antibodies induced by MCS-Rv1768-Rv1983 markedly blocking MTB invasion into macrophages. This antibody-mediated ‘neutralizing’ effect is distinct from that of classical opsonophagocytosis. We hypothesize that by preventing MTB from entering its preferred intracellular niche, bacteria remain extracellular where they become susceptible to a broader array of host defences. First, extracellular bacteria are more readily recognized and lysed by natural killer (NK) cells and complement components. Second, compared with mannose receptor-mediated entry exploited by MTB, antibody-opsonized bacteria can be captured by macrophages via Fcγ receptors, which may trigger a more robust pro-inflammatory response and enhanced phagosome lysosomal fusion. Thus, the present strategy does not simply bypass macrophages; rather, it redirects the pathogen to a more destructive phagocytic route while also exposing it to extracellular immune effectors. This dual-mechanism prevention of invasion (humoral) and the killing of intracellular bacteria (cellular) represent a paradigm shift from the traditional focus solely on cellular immunity and highlights the potential of this vaccine strategy.

Nanocarrier vaccines, as immunological adjuvants, can increase the immunogenicity of protein antigens (36). Chitosan is the most widely used nanoadjuvant material, but further improvements are still needed before its clinical application (37). Macrophages express mannose receptors on their surface; thus, mannose-modified chitosan nanoparticles are more likely to be engulfed by macrophages, thereby triggering an immune response (38). Studies have shown that tri-mannose-grafted chitosan nanoparticles affect mitochondrial structure, increase cellular metabolism and upregulate the expression of numerous immune-related genes in macrophages (39,40). In the present study, the synergistic effect of MCS and Rv1768-Rv1983 likely stems from i) targeted delivery to mannose receptors on APCs, promoting efficient antigen uptake and cross-presentation and ii) the intrinsic immunostimulatory properties of chitosan, which promote APC maturation and cytokine release, creating a favourable milieu for T-cell polarization. This dual action explains the superior antibody and T-cell responses observed with MCS-encapsulated antigen compared with the protein alone.

For a long time, the anti-TB immunity has been focussed mainly on cellular immunity, particularly the cytotoxic effect induced by CD8^+^ T cells (41); however, the anti-TB effect of antibodies has not received sufficient attention. In the present study, a novel subunit vaccine for TB based on the Rv1768-Rv1983 fusion protein and MCS nanoparticles was successfully developed and evaluated. This vaccine not only elicits a balanced Th1/Th2 mixed immune response but also uniquely induces the production of antibodies with ‘neutralizing activity’, effectively blocking the initial invasion of macrophages by MTB. Bacteria that do not enter macrophages are more likely to be eliminated by NK cells and other extracellular mechanisms (42). In addition, the presence of antibodies alters the way macrophages engulf bacteria, which can activate the complement system and inhibit the immune escape of MTB associated with macrophage inflammation and glycolytic activation (43,44). This mechanism offers a new direction for TB vaccine development: Shifting from the traditional ‘enhanced bactericidal’ strategy to a preventive ‘block invasion’ approach.

From a translational research perspective, the present study validates the use of MCS as a versatile and robust platform for delivering complex PE/PPE antigens, which are notoriously difficult to handle. Given that the MTB genome encodes a large number of these immunogenic proteins, the present work establishes a replicable methodological pipeline. Researchers can adapt this MCS nanocapsule system to test other combinations of MTB antigens, particularly those with poor solubility or those that require specific targeting to APCs. The present proof-of-concept study thus provides a valuable tool for accelerating the discovery of next-generation subunit vaccines against TB.

There are a few limitations to acknowledge. First, the degree of mannose substitution on chitosan was not measured in the exact batches used for the animal experiments because of sample depletion; however, the protocol was based on well-established methods, and FTIR confirmed successful conjugation. Second, the sample size for survival analysis was based on a power calculation, but larger cohorts would further strengthen the findings. Third, while the present flow cytometry gating strategy is a well-established method consistent with our previous work (45), the absence of anti-CD3 staining could be considered a technical limitation. Future studies will include this additional marker to further validate T-cell identification. Future studies should also investigate the long-term durability of the immune response and the protective efficacy against different MTB strains.

In conclusion, the present findings demonstrate that the MCS-Rv1768-Rv1983 nanovaccine is a highly promising candidate for TB prevention, warranting further in-depth preclinical and clinical studies.

## Supplementary Material

TMHMM 2.0 tool was used to predict the transmembrane regions of the (A) Rv1768 and (B) Rv1983 proteins respectively.

Effects of different MCS/TPP mass ratios on the (A) size and (B) zeta potential of MCS. MCS, mannosylated chitosan; TPP, tripolyphosphate sodium.

Effects of pH of different MCS solutions on the (A) size and (B) zeta potential of MCS. MCS, mannosylated chitosan.

The effects of different concentrations of fusion proteins on drug loading of protein mannosylated chitosan nanoparticles.

Representative flow cytometry plots corresponding to Fig. 4. (A) Gating strategy: Lymphocytes were first gated based on FSC and SSC, followed by gating on CD4^+^ or CD8^+^ T cell populations. (B) Representative plots of IFN-γ^+^ and IL-4^+^ CD4^+^ T cells for each experimental group [Control (PBS), MCS, Rv1768-Rv1983 and MCS-Rv1768-Rv1983]. (C) Representative plots of IFN-γ^+^ and IL-4^+^ CD8^+^ T cells for each group. The percentages of positive cells are indicated in each plot. Peripheral blood mononuclear cells were isolated from mice 2 weeks after the first immunization and stimulated *ex vivo* with the Rv1768-Rv1983 fusion protein (5 *μ*g/ml) for 16 h. Cells were surface-stained with anti-CD4 or anti-CD8 antibodies and intracellularly stained with anti-IFN-γ and anti-IL-4 antibodies. FSC, forward scatter; SSC, side scatter; MCS, mannosylated chitosan.

## Figures and Tables

**Figure 1:**
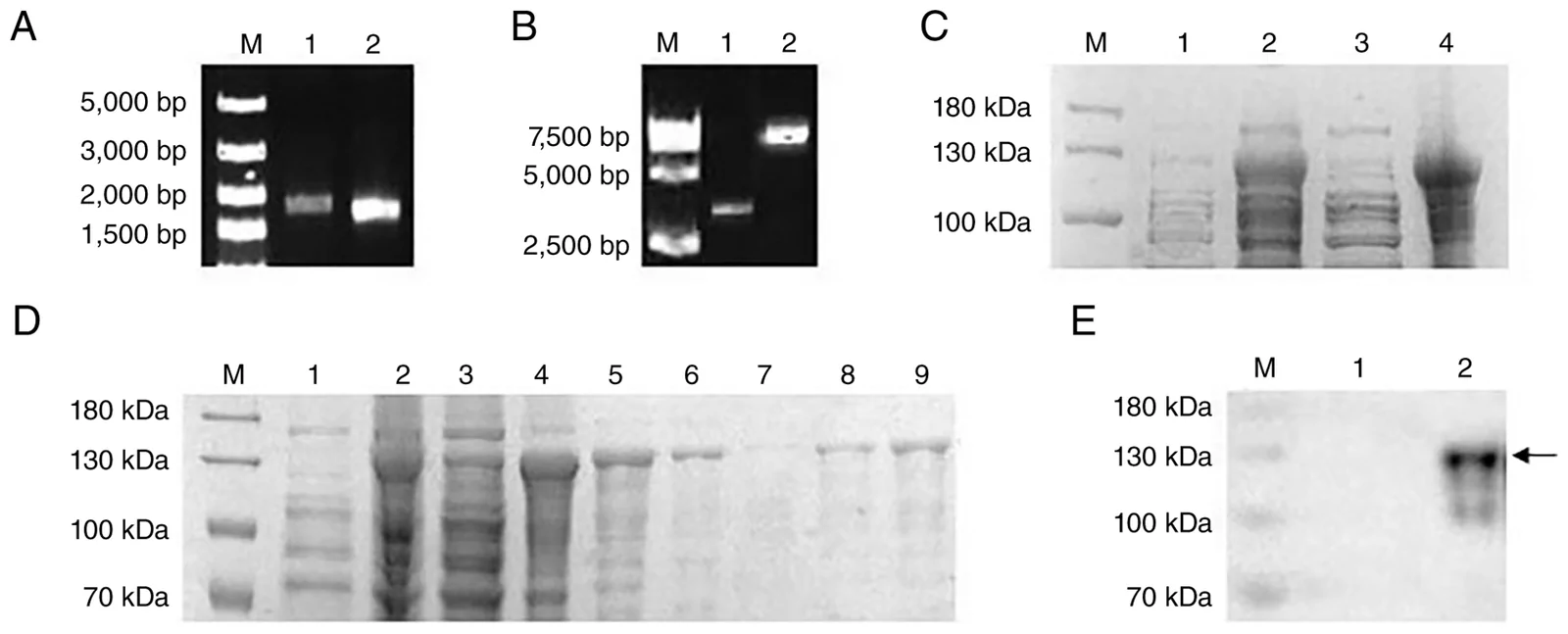
Expression and purification of Rv1768-Rv1983 fusion protein. (A) PCR amplification of Rv1768 gene (lane 1, 1,854 bp) and Rv1983 gene (lane 2, 1,674 bp). (B) Overlap-PCR amplification of Rv1768-Rv1983 fusion gene (lane 1) and recombinant vector pET32a-Rv1768/Rv1983 (lane 2). (C) SDS-PAGE analysis of fusion protein expression. Lane M: Protein marker (kDa); lane 1: Before induction; lane 2: After induction; lane 3: Supernatant after bacterial lysis; lane 4: Pellet after lysis (inclusion bodies). (D) Purification by nickel-nitrilotriacetic acid affinity chromatography. Lane 1 was the sample supernatant before induction; lane 2 was the sample inclusion body before induction; lane 3 was the sample supernatant after induction; lane 4 was the sample inclusion body after induction; lane 5 was flow-through liquid after overnight incubation of protein and filler; lanes 6-9: Elution with 20, 80, 100 and 150 mM imidazole. (E) Western blot confirmation using anti-His tag antibody. Lane 1: Empty vector control; lane 2: Purified fusion protein (arrow, ~129 kDa).

**Figure 2:**
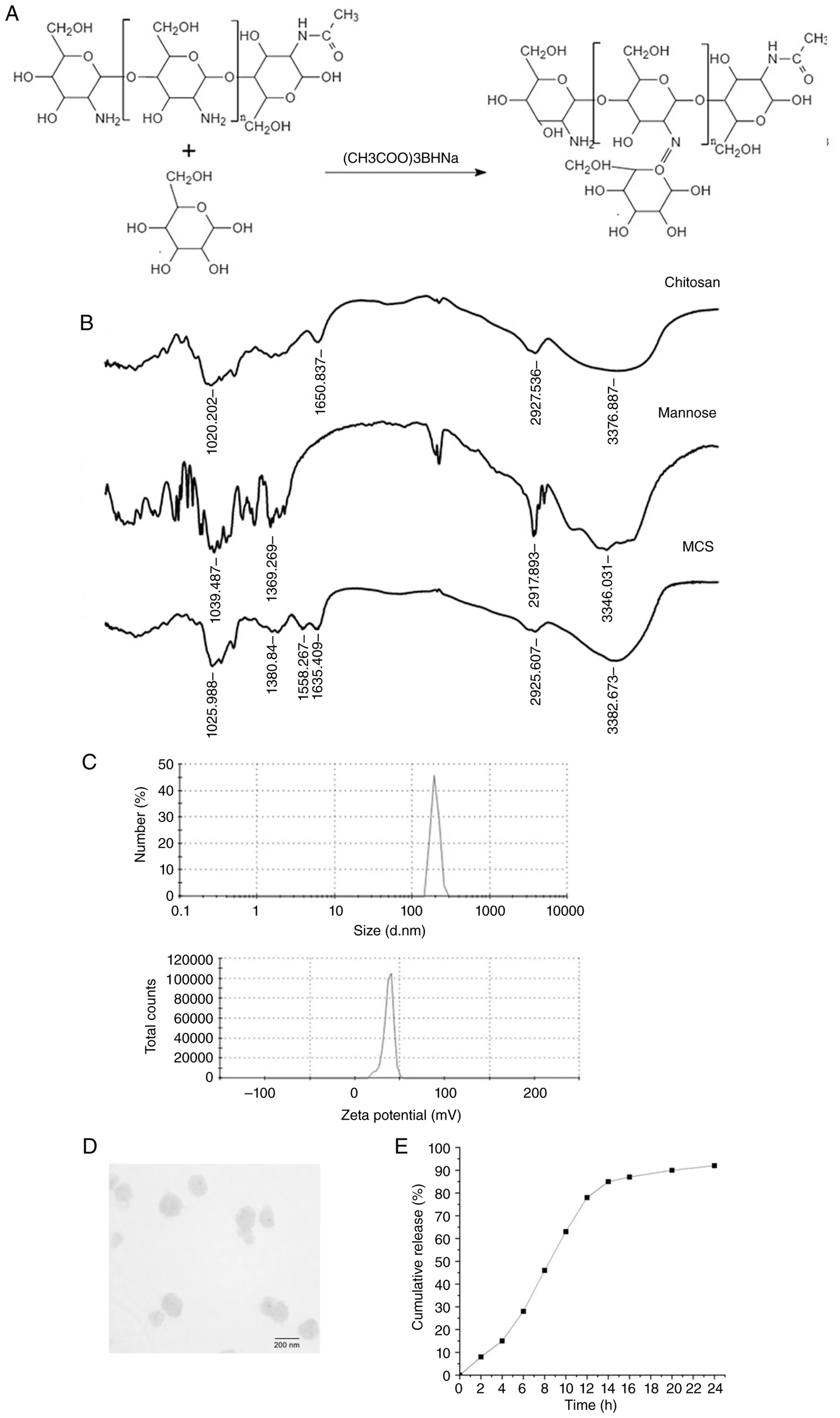
Characterization of MCS-Rv1768-Rv1983 nanoparticles. (A) Synthetic route of MCS. (B) Fourier transform infrared spectroscopy spectra of mannose, chitosan and MCS. (C) Particle size distribution (upper) and zeta potential (lower) of the nanoparticles. (D) Transmission electron microscopy image (scale bar, 200 nm). (E) *In vitro* cumulative release profile of fusion protein from nanoparticles in PBS (pH 7.4) at 37˚C; representative release profile from three independent experiments with similar results. MCS; mannosylated chitosan.

**Figure 3:**
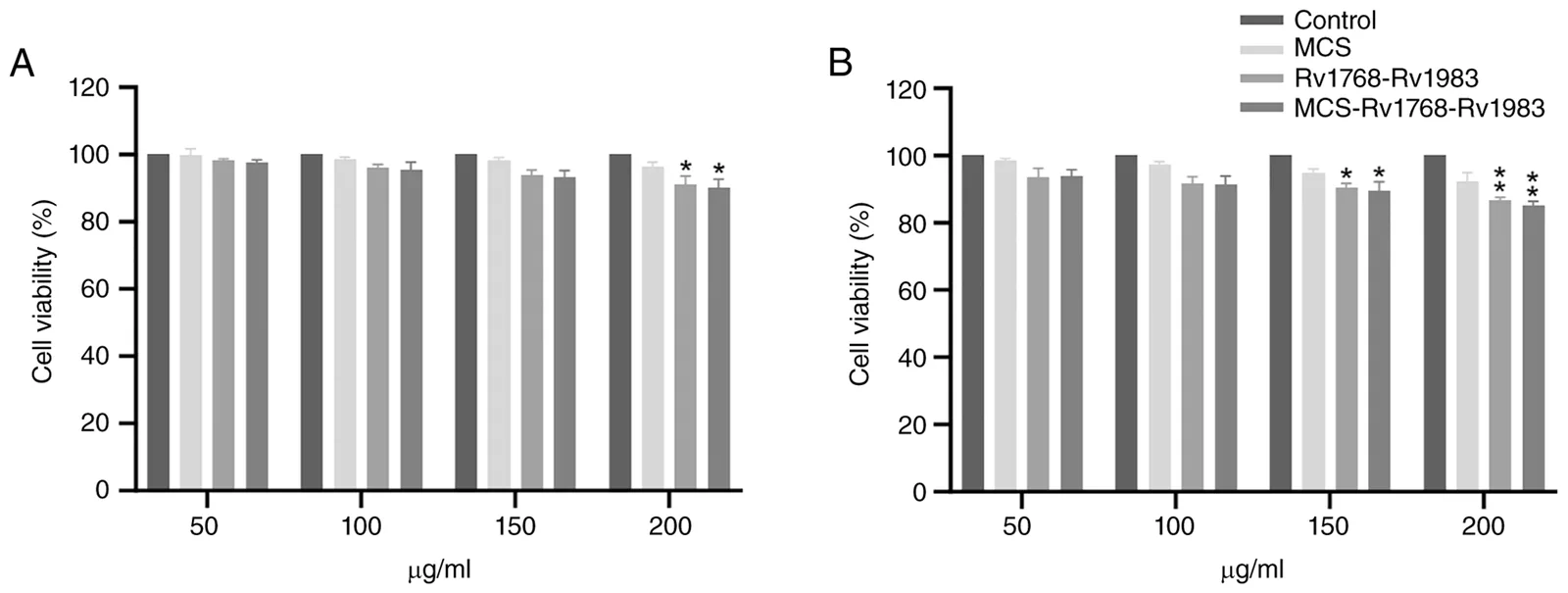
Cytotoxicity of MCS, free Rv1768-Rv1983 and MCS-Rv1768-Rv1983 on THP-1 cells. Cells were treated with indicated concentrations (50-200 µM) for (A) 24 and (B) 48 h. Cell viability was measured using a Cell Counting Kit-8 assay. Data are presented as mean ± SEM (n=3). One-way ANOVA with Student-Newman-Keuls post hoc test was used to compare differences among concentration groups at each time point; ^*^P<0.05 vs. PBS control. Viability >70% is considered non-cytotoxic according to ISO 10993-5. MCS; mannosylated chitosan.

**Figure 4:**
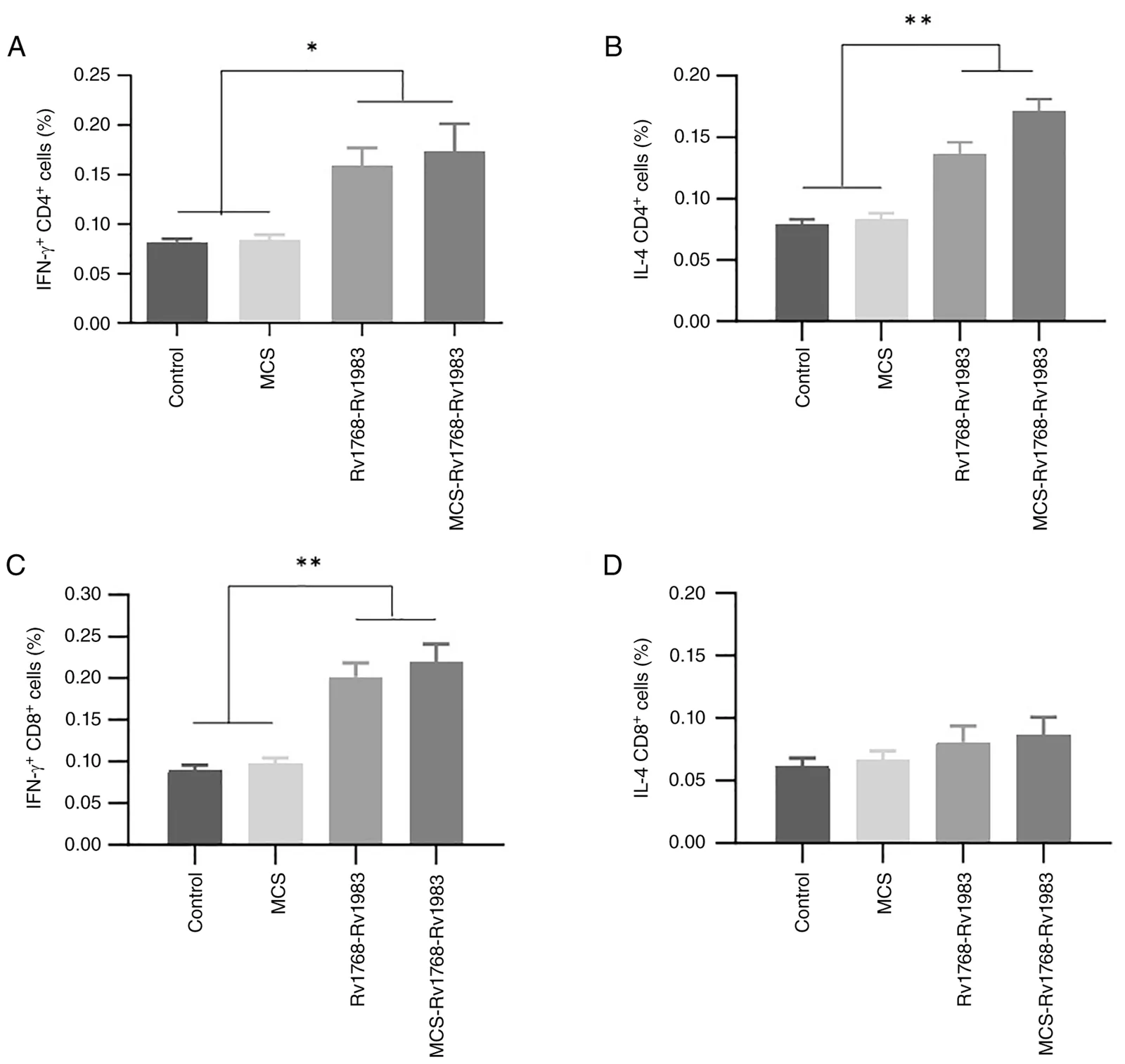
Cytokine-producing T cell populations in peripheral blood mononuclear cells 2 weeks after first immunization. (A) Percentage of IFN-γ^+^ CD4^+^ T cells. (B) Percentage of IL-4^+^ CD4^+^ T cells. (C) Percentage of IFN-γ^+^ CD8^+^ T cells. (D) Percentage of IL-4^+^ CD8^+^ T cells. Data are presented as mean ± SEM (n=6). One-way ANOVA with Student-Newman-Keuls post hoc test; ^*^P<0.05, ^**^P<0.01. Representative flow cytometry plots showing the gating strategy and cytokine-positive populations for each group are provided in Fig. S5. MCS; mannosylated chitosan.

**Figure 5:**
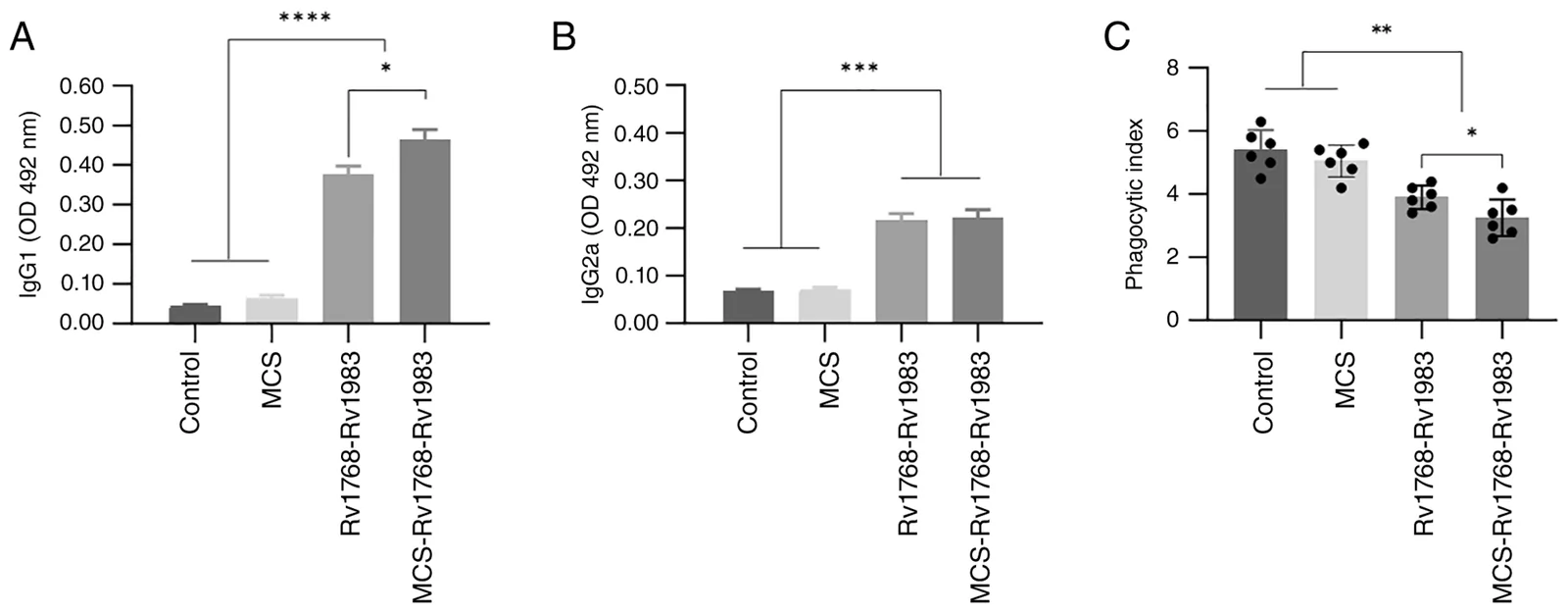
Antibody responses and inhibition of macrophage invasion. (A) Serum IgG1 titers. (B) Serum IgG2a titers. (C) Phagocytic index of RAW264.7 macrophages after incubation with H37Rv opsonized with sera from different immunization groups. Data are mean ± SEM (n=6). One-way ANOVA with Student-Newman-Keuls post-hoc test; ^*^P<0.05, ^**^P<0.01, ^***^P<0.001, ^****^P<0.0001. MCS; mannosylated chitosan.

**Figure 6:**
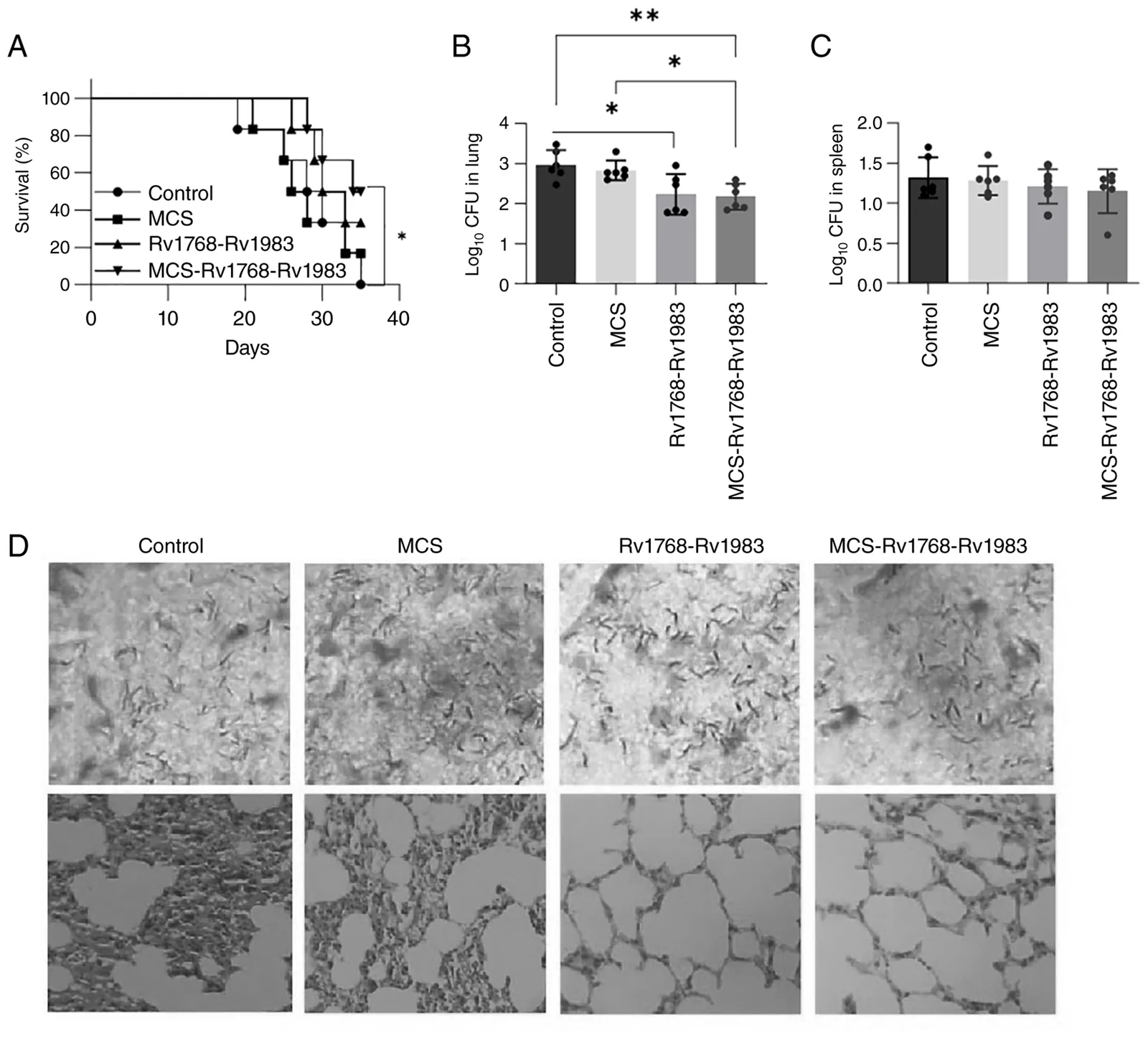
*In vivo* protective efficacy against H37Rv challenge. (A) Kaplan-Meier survival curves (n=6 per group, log-rank test). (B) Bacterial loads (log_10_ CFU) in lungs. (C) Bacterial loads in spleens. (D) Acid-fast staining (upper panels; magnification, x1,000) and H&E staining (lower panels; magnification, x40) of lung sections. Data for CFU are presented as mean ± SEM (n=6). One-way ANOVA with Student-Newman-Keuls post hoc test; ^*^P<0.05, ^**^P<0.01. MCS; mannosylated chitosan.

## Data Availability

The data generated in the present study may be requested from the corresponding author.
