# Supplementary material for: Targeted inhibition of Mycobacterium tuberculosis invasion into macrophages by Rv1768-Rv1983-loaded mannosylated chitosan nanoparticles
Source: Exp Ther Med. 2026 Oct 15;32(6):342. doi: 10.3892/etm.2026.13337 (PMC13711149; doi:10.3892/etm.2026.13337)

Figure S1. TMHMM 2.0 tool was used to predict the transmembrane regions of the (A) Rv1768 and (B) Rv1983 proteins respectively.

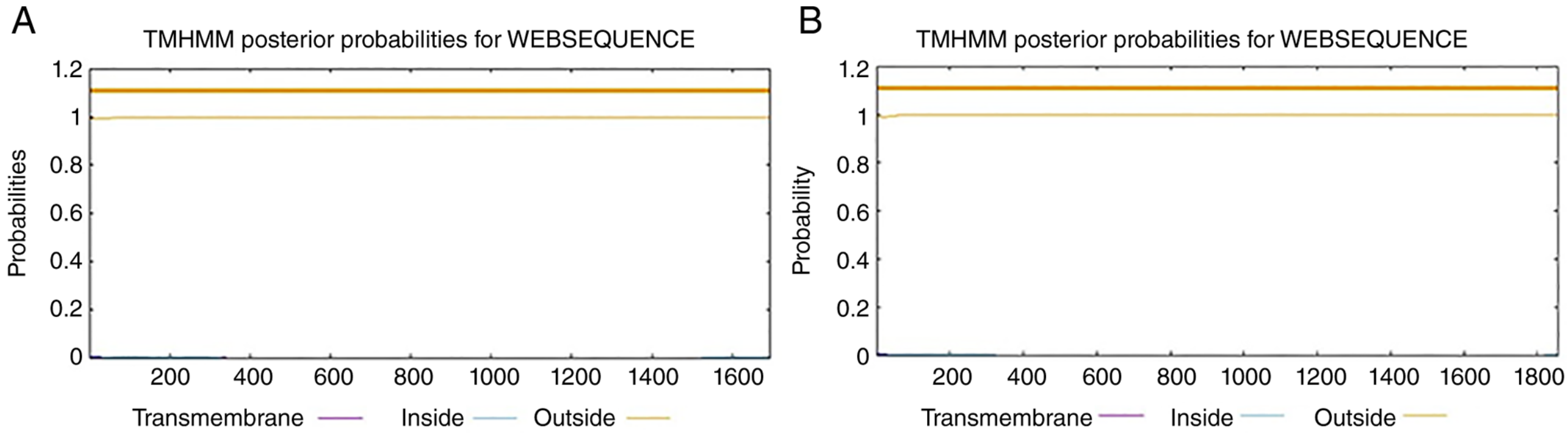

Figure S2. Effects of different MCS/TPP mass ratios on the (A) size and (B) zeta potential of MCS. MCS, mannosylated chitosan; TPP, tripolyphosphate sodium.

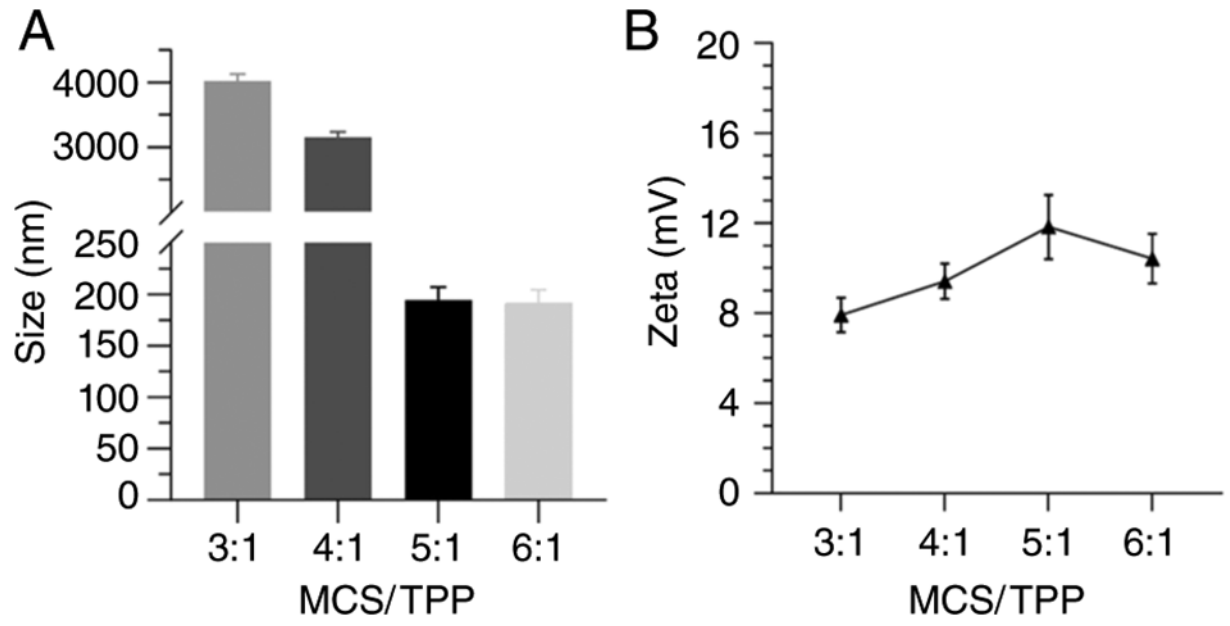

Figure S3. Effects of pH of different MCS solutions on the (A) size and (B) zeta potential of MCS. MCS, mannosylated chitosan.

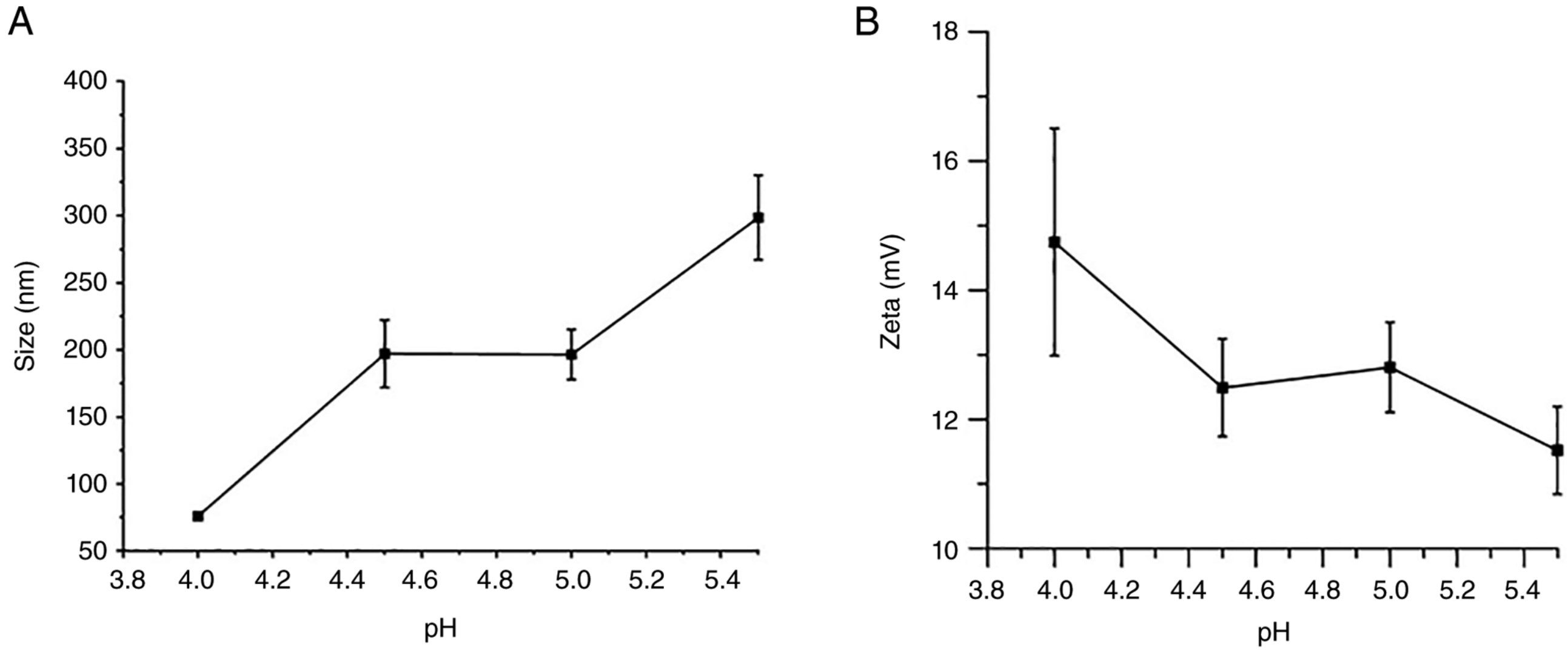

Figure S4. The effects of different concentrations of fusion proteins on drug loading of protein mannosylated chitosan nanoparticles.

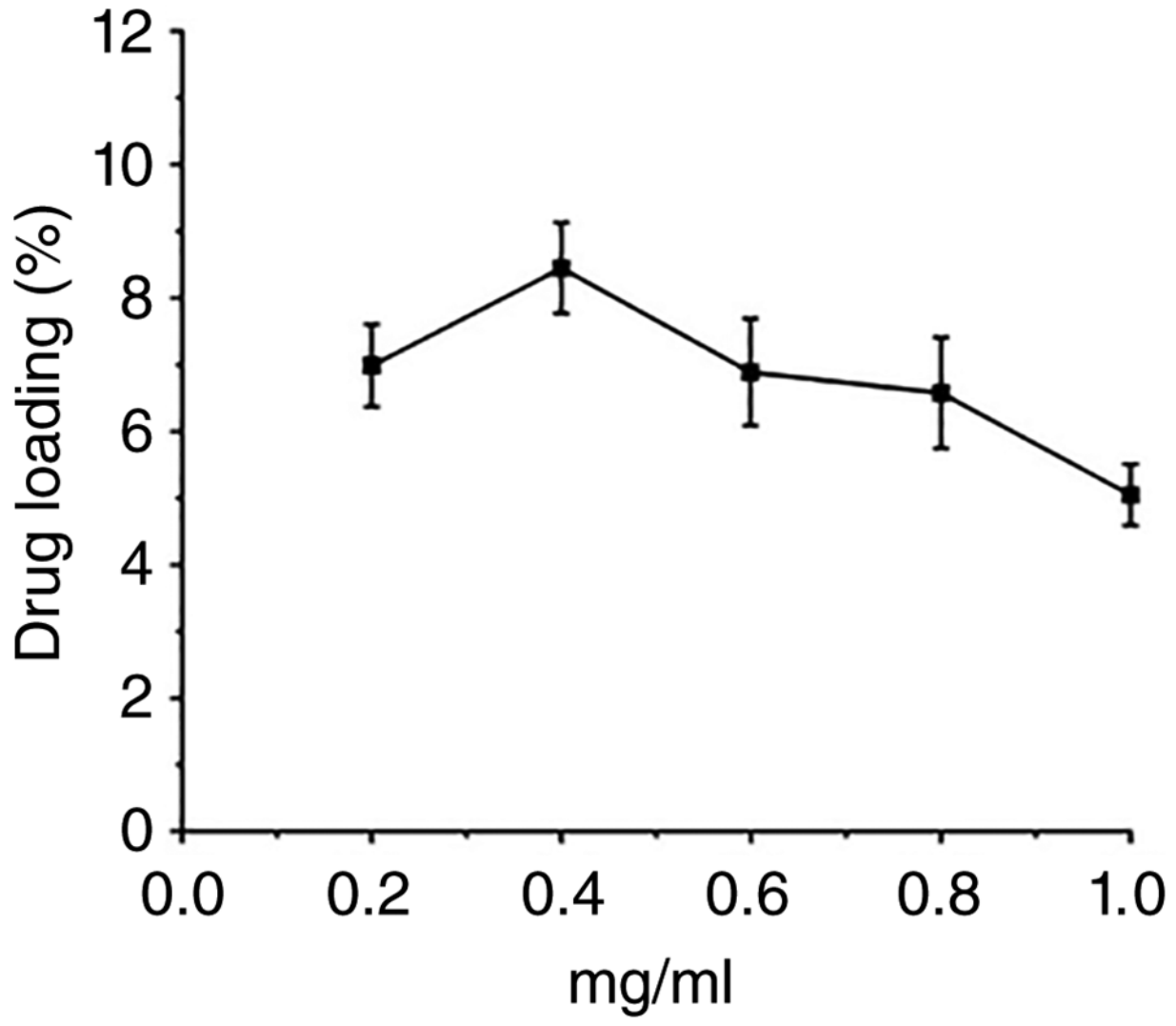

Figure S5. Representative flow cytometry plots corresponding to Fig. 4. (A) Gating strategy: Lymphocytes were first gated based on FSC and SSC, followed by gating on $CD4^+$ or $CD8^+$ T cell populations. (B) Representative plots of IFN-γ^+ and IL-4^+ $CD4^+$ T cells for each experimental group [Control (PBS), MCS, Rv1768-Rv1983 and MCS-Rv1768-Rv1983]. (C) Representative plots of IFN-γ^+ and IL-4^+ $CD8^+$ T cells for each group. The percentages of positive cells are indicated in each plot. Peripheral blood mononuclear cells were isolated from mice 2 weeks after the first immunization and stimulated *ex vivo* with the Rv1768-Rv1983 fusion protein (5 μg/ml) for 16 h. Cells were surface-stained with anti-CD4 or anti-CD8 antibodies and intracellularly stained with anti-IFN-γ and anti-IL-4 antibodies. FSC, forward scatter; SSC, side scatter; MCS, mannosylated chitosan.

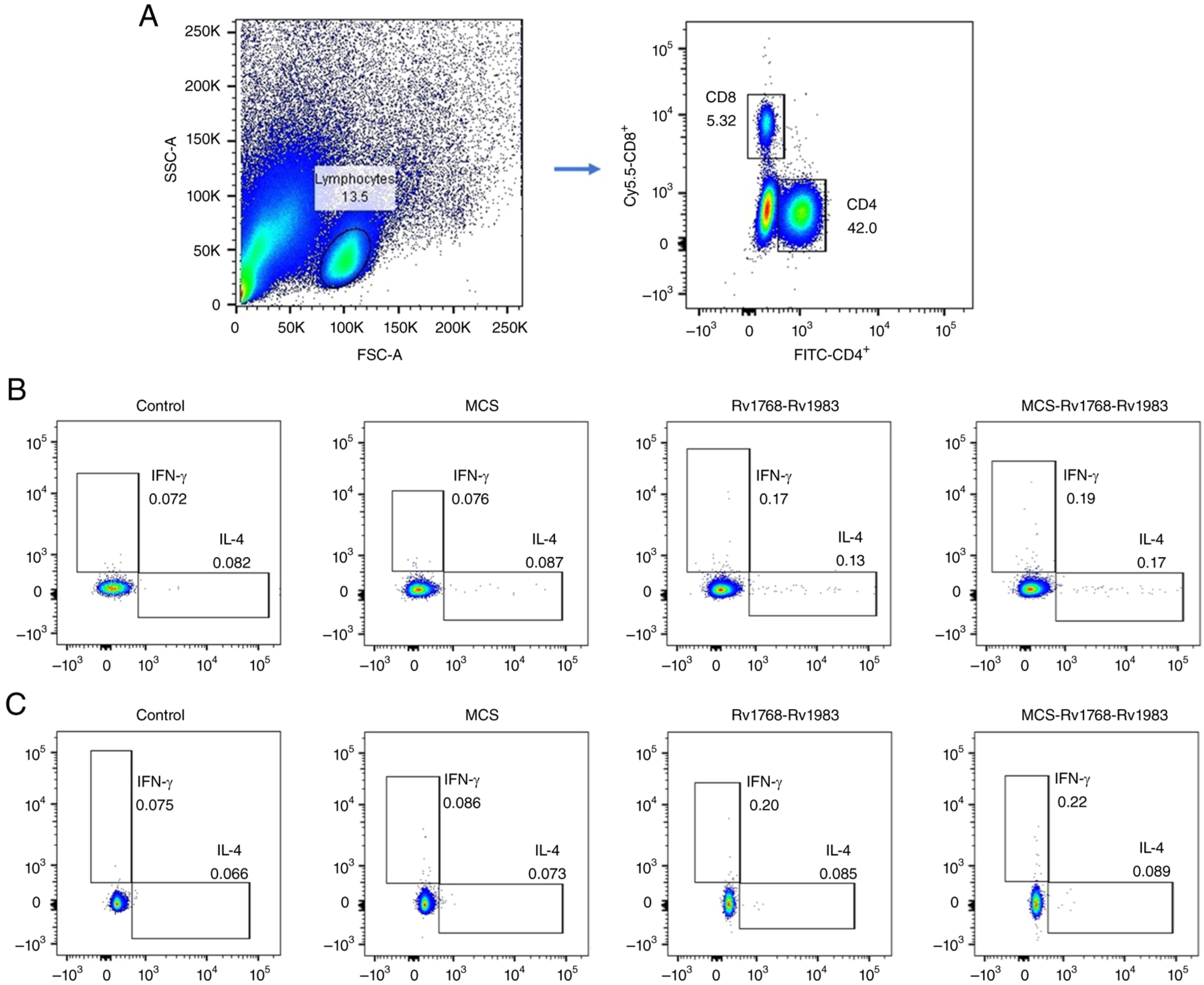

Supplement: Supplementary file 1 [file Supplementary_Data.pdf]
